# Cytoskeletal assembly in axonal outgrowth and regeneration analyzed on the nanoscale

**DOI:** 10.1038/s41598-022-18562-5

**Published:** 2022-08-23

**Authors:** Max Hofmann, Lucas Biller, Uwe Michel, Mathias Bähr, Jan Christoph Koch

**Affiliations:** Department of Neurology, University Medical Center Göttingen, Göttingen, Germany

**Keywords:** Development of the nervous system, Regeneration and repair in the nervous system

## Abstract

The axonal cytoskeleton is organized in a highly periodic structure, the membrane-associated periodic skeleton (MPS), which is essential to maintain the structure and function of the axon. Here, we use stimulated emission depletion microscopy of primary rat cortical neurons in microfluidic chambers to analyze the temporal and spatial sequence of MPS formation at the distal end of growing axons and during regeneration after axotomy. We demonstrate that the MPS does not extend continuously into the growing axon but develops from patches of periodic βII-spectrin arrangements that grow and coalesce into a continuous scaffold. We estimate that the underlying sequence of assembly, elongation, and subsequent coalescence of periodic βII-spectrin patches takes around 15 h. Strikingly, we find that development of the MPS occurs faster in regenerating axons after axotomy and note marked differences in the morphology of the growth cone and adjacent axonal regions between regenerating and unlesioned axons. Moreover, we find that inhibition of the spectrin-cleaving enzyme calpain accelerates MPS formation in regenerating axons and increases the number of regenerating axons after axotomy. Taken together, we provide here a detailed nanoscale analysis of MPS development in growing axons.

## Introduction

The cytoskeleton plays a crucial role in the outgrowth and regeneration of axons. It enables the neuron to extend its axon over very long distances of up to one meter. The cytoskeleton must withstand mechanical force and torsion but also needs to facilitate adaption and response to trauma or damage, like axonal lesions. Moreover, it builds the scaffold for the precise distribution of membrane channels and receptors along the axon and the basis for axonal transport, which are both essential for the function of the neuron.

Recent advances in super-resolution microscopy (SRM) have opened up a door for nanoscale observations of biological structures. In light of these advances, Xu et al. found that the cytoskeletal proteins actin, spectrin, and adducin form highly periodic structures in the axon, the membrane-associated periodic skeleton (MPS)^1^. Originally discovered using stochastic optical reconstruction microscopy (STORM), this periodic arrangement was soon confirmed using other SRM methods like stimulated emission depletion (STED) microscopy^2^.

The MPS is prevalent in a broad range of neuronal cell types across different species^3,4^. It has also been shown in brain slices^1^ and in living neurons^5^. Several studies have highlighted a broad range of involvements for the MPS in neuronal processes, like stabilization of axons^6^, electrical conductivity^7^, lattice-like organization of proteins^1,8–10^, and control of axonal diameter^7,11^. It was established that the MPS consists of actin rings, made up of actin filaments, capped by adducin, interconnected by α- and β-spectrin tetramers^1,11^.

Several studies have suggested that the development of the MPS within the growing axon occurs in a proximal to distal pattern^12–14^. The MPS starts to form close to the soma at an early stage during axon development, at day in vitro (DIV) 2 in hippocampal rat neurons^12^. It then develops and progresses along the growing axon over time. Interestingly, in neural stem cells, short periodic patches of a 1D arrangement of actin and spectrin were reported in the soma^15^. These neurons showed a mature MPS once they had matured.

The role of the MPS in axonal degeneration has been studied previously^16,17^. It was shown that axonal degeneration induced by trophic factor withdrawal led to a loss of the MPS^16,17^, but this was independent of caspase apoptotic pathways^17^. Interestingly, stabilization of F-actin using the drug cucurbitacin-E preserved the MPS and reduced axonal degeneration^16^. In the last decade, mechanical axotomy through vacuum media aspiration in neurons cultured in microfluidic systems has proven to be a reliable model to study axonal degeneration and regeneration in vivo^18,19^.

Despite this recent interest in the MPS and its development, the exact sequence and mechanism by which the MPS extends to newly formed parts of the outgrowing or regenerating axon remain unknown. Here, we provide a detailed nanoscale analysis of the MPS development in growing axons and the cytoskeletal changes upon regeneration after axotomy. We found that growing axons display a characteristic spatial and temporal increase of spectrin periodicity in a gradient from the proximal axonal regions towards the distal growth cone. We demonstrate that the building blocks in the formation of the MPS are periodic arrangements of βII-spectrin (periodic patches) that grow in size and number and eventually coalesce. In regenerating axons, the MPS develops faster and at more distal axon regions than physiologically outgrowing axons.

## Results

### Distribution of spectrin and tubulin along the axon and the growth cone in rat cortical neurons

**Figure 1:**
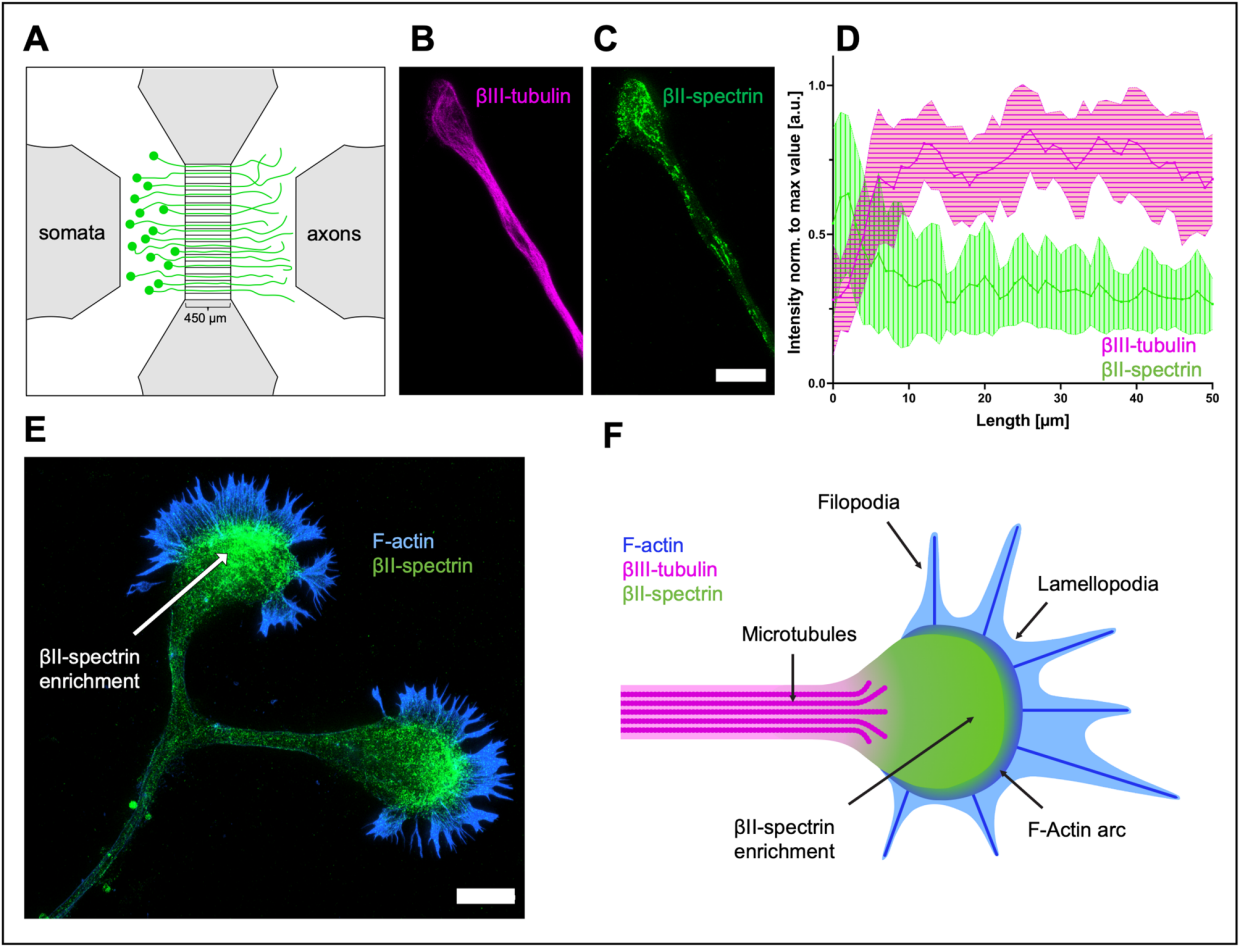
Distribution of βII-spectrin and βIII-tubulin along the axon and the growth cone in non-axotomized rat cortical neurons. (**A**) Schematic drawing of a microfluidic chamber seeded with primary cortical neurons in the somatic department, transduced with AAV.EGFP virus. These neurons extend their axons through the microgrooves (length: 450 µm) into the axonal compartment within 11 days (green cells). (**B** and **C**) STED images of cortical neurons stained for spectrin and tubulin. Note that spectrin is enriched in the growth cone while tubulin concentration is decreased. Scale bar 5 µm. (**D**) Quantification of line intensity scans along the axon, starting from the growth cone tip. Spectrin fluorescence intensity peaks at the growth cone, whereas the tubulin signal is lower compared to the axon. Data is represented as mean ± SD. At least 5 biological replicates were analyzed, 11 axons were analyzed in total. (**E**) Representative STED image of a growth cone stained for spectrin and F-actin. Spectrin is enriched close to the actin filaments. Scale bar 5 µm. (**F**) Schematic drawing of a growth cone, showing an enrichment of βII-spectrin in the apical central domain of the GC and microtubules splaying into the GC. The depicted F-actin arc resembles the transition domain, whereas microtubules splay into the central domain of the GC.

To analyze the organization of the developing MPS in physiologically outgrowing (non-axotomized) and in regenerating (after axotomy) axons, we cultured primary rat embryonic cortical neurons in compartmentalized microfluidic chambers^19^ (Fig. 1A). The neurons were seeded in the soma compartment and extended their axons through microgrooves (length 450 µm, width 3 µm) to the axonal compartment allowing the separate analysis of axons. Cells were fixed on day 11, immunostained against different cytoskeletal proteins, and analyzed using STED microscopy.

First, we studied the general spatial distribution of essential components of the axonal cytoskeleton along the non-axotomized axon. We performed line intensity scans for βII-spectrin and βIII-tubulin along the growth cone (GC) and the adjacent axon (Fig. 1, B–D, Supplemental Fig. 1). As methanol fixation employed here extracts most of the soluble tubulin, the tubulin signal should represent preserved microtubules^20^. The intensity of tubulin was highest along the distal axon shaft and significantly lower in the GC by 40% (0.44 ± 0.04 vs. 0.75 ± 0.02), which might at least partly be due to the splaying of microtubules in the GC. In contrast, the intensity of spectrin was highest in the GC and was 65% lower along the axon (0.5 ± 0.04 vs. 0.3 ± 0.04). Both spectrin and tubulin intensity showed constant values further proximal up to 250 µm from the GC. We thus conclude that spectrin is strongly enriched in the GC and integrated into the axonal cytoskeleton in a lower concentration.

The spectrin signal was strongest in the apical central domain of the growth cone. In GCs co-stained for spectrin and F-actin, spectrin was often enriched in the proximity to actin filaments, as can be seen in Supplemental Figs. 2, 3. Figure 1E,F illustrate the location of the enrichment of spectrin in the GC relative to the F-actin-dominated filopodia. Interestingly, in some axonal GCs, at the base of F-actin bundles, the spectrin signal was enhanced and colocalized with actin. Exemplary micrographs are shown in Supplemental Fig. 3.

**Figure 2:**
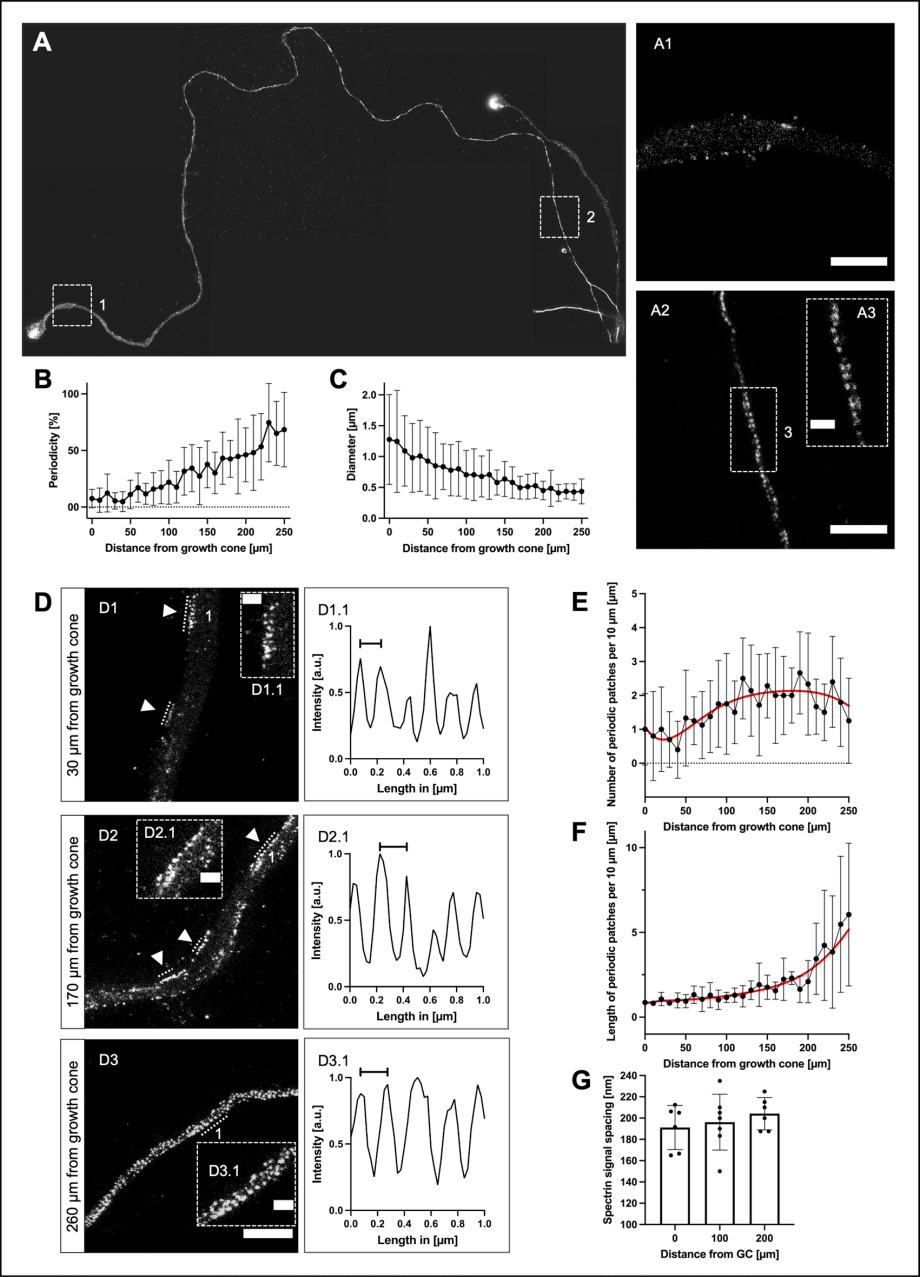
Development of the membrane-associated periodic skeleton along the growing axon. (**A**) Representative image of an axon with its growth cone, stained for spectrin, stitched from multiple STED images, up to 270 µm towards soma. **A1** shows an enlarged axon section at 10 µm distance from the growth cone, no periodicity is visible (scale bar 2 µm). **A2** shows an enlarged section of **A**, 270 µm from the growth cone (scale bar 2 µm). **A3** shows an enlarged section of **A2**, MPS is clearly visible (scale bar 0.5 µm). (**B**) Graphical display of the development of periodicity over the length of the axon, starting from the growth cone base. Periodicity is increasing linearly towards the soma. Data is represented as mean ± SD. At least 5 biological replicates were analyzed, 11 axons were analyzed in total. Because of limitations in following axons back for extended lengths, not all axons could be included at distal points. (**C**) Reduction of the axonal diameter over the length of the axon, starting from the growth cone base, contrariwise to (**B**). Data is represented as mean ± SD. At least 5 biological replicates were analyzed, 11 axons were analyzed in total. Because of limitations in following axons back for extended lengths, not all axons could be included at distal points. (**D**) Three sections of an axon stained for spectrin, showing different stages of MPS development, were imaged using STED microscopy. **D1** is 30 µm afar from the growth cone, **D2** 170 µm, and **D3** 260 µm. The arrowheads point to periodic patches, periodic arrangements of spectrin with at least three signals (scale bar 2 µm). **D1.1** shows the periodic plot of a line intensity scan along the numbered periodic patch in **D1**. The marked distance is 150 nm. **D2.1** shows a periodic line intensity scan along the numbered periodic patch in **D2**. The marked distance is 200 nm. **D3.1** shows the periodic plot of a line intensity scan along the numbered periodic patch in **D3**. The marked distance is 200 nm. The scale bar length of the enlarged images **D1.1**, **D2.1**, and **D3.1** is 0.5 µm each. (**E** and **F**) Quantification of parameters for the development of MPS along the axon, starting from the growth cone base. The number of periodic patches (**E**) increases, then decreases beyond 200 µm. The length of periodic patches (**F**) increases towards soma. Data is represented as mean ± SD. Red lines indicate estimated curve fits. At least 5 biological replicates were analyzed, 10 axons were analyzed in total. Because of limitations in following axons back for extended lengths, not all axons could be included at distal points. (**G**) Development of the spacing of spectrin tetramers in periodic patches in 10 µm long axonal segments at a distance of 0 µm, 100 µm, and 200 µm from the growth cone in non-axotomized axons. Although a slight increase of spectrin tetramer spacing along the axon is visible, no significant changes were detected using ordinary one-way ANOVA, followed by Turkey’s multiple comparisons test. Bars represent mean ± SD, at least 5 biological replicates were analyzed, 11 axons were analyzed in total.

**Figure 3:**
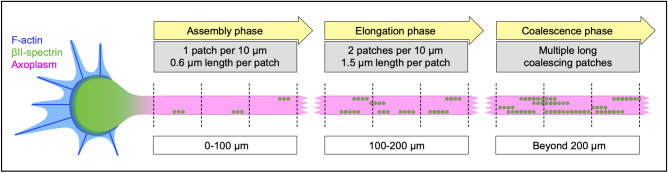
Phases of MPS development along the growing axon. Schematic drawing of the phases of MPS development along the growing axon, the growth cone is to the left. The dotted lines mark 10 µm intervals. βII-Spectrin molecules form periodic patches (symbolized by small green circles) close to the growth cone (*assembly phase*). These patches then increase in number and size *(elongation phase)*, coalesce during maturation, and form the final MPS (*coalescence phase*).

### Development of the membrane-associated periodic skeleton in non-axotomized rat cortical neurons

Zhong et al. reported that the MPS of hippocampal neurons starts to develop near the soma and matures along the axon towards the GC^12^. To better understand the process of MPS assembly in a growing axon, we asked how the MPS develops in the youngest part of the axon, i.e., the segment adjacent to GC. We, therefore, analyzed the first 250 µm of the axonal segment close to the GC (Fig. 2A). We divided the axon into 10 µm segments and evaluated for each respective segment the total length of the segment showing a periodic arrangement of spectrin with a distance of around 190 µm between spectrin signals. The respective length of axon showing MPS per respective segment was then divided by 10 µm. As depicted in Fig. 2, B, there was a consistent, nearly tenfold increase of axonal spectrin periodicity from the axonal GC base up to 250 µm towards the soma (first 10 µm: 7.5% ± 2.4%, 250 µm from GC: 68.5% ± 16.5%). A periodicity of 50% (5/10 µm of measured axon showed periodicity) was reached at a mean distance of 220 µm from the GC. With an average growth speed of 18.3 µm/h in our culturing conditions, this distance corresponds to a maturation time of 12 h for this axonal segment. A fully developed MPS (10/10 µm measured axon showed periodicity) was seen beyond distances of 250–350 µm from the GC. This distance corresponds to a maturation time of approximately 14–19 h until an axonal segment contains a continuous MPS.

A role of the MPS in influencing the axonal diameter has been described in previous studies^7,11^. We, therefore, analyzed the development of the axonal diameter along the axon. As Fig. 2C shows, there was a nearly three-fold decrease of axonal diameter along a distance of 250 µm from the GC (first 10 µm: 1.3 µm ± 0.2 µm, 250 µm from GC: 0.4 µm ± 0.1 µm). The finding that the youngest part of the axon shows the lowest periodicity, but greatest diameter, supports the idea of a progressive constriction of the axonal diameter by the MPS^11^.

### The MPS develops in periodic patches

We noticed that spectrin periodicity does not extend continuously from proximal to distal but develops in the distal axon from multiple periodic spectrin patches extending and merging towards the soma (Fig. 2D). A “periodic spectrin patch” was defined as a continuous array of at least three spectrin-fluorescence signals inside the axon with a mean periodic distance of 150–250 nm. This distance was chosen based on the literature^1,21^ and to take possible changes of spectrin tetramer spacing during maturation into account. The number and length of these periodic spectrin patches were measured in 10 µm segments along the growing axon starting at the base of the GC.

Short periodic spectrin patches appeared in the first 100 µm adjacent to the GC, with a density of 1 patch per 10 µm of axon (1.1 ± 0.1). The density increased to 2 patches per 10 µm between 100 and 200 µm from the GC (2.1 ± 0.1) and then slowly decreased again at distances > 200 µm from the GC as single periodic spectrin patches extended and coalesced (1.8 ± 0.6 at 250 µm) (Fig. 2E).

The mean length of the periodic spectrin patches in the first 100 µm adjacent to the GC was 0.6 µm (644 nm ± 96.4 nm), corresponding to 3 consecutive spectrin tetramers. Between 100 and 200 µm from the GC, the length of the periodic spectrin patches increased gradually (1525 nm ± 14.7 nm) and then exponentially beyond 200 µm to a mean length of 5.5 µm (5476 nm ± 179.6 nm) at 250 µm, corresponding to around 27 spectrin tetramers (Fig. 2F).

The average spacing of spectrin tetramers along the axon slowly increased from around 190 nm at the GC-base to 200 nm towards the soma (10 µm: 191.2 nm ± 8.5 nm, 100 µm: 196.2 nm ± 9.9 nm, and 200 µm from the GC: 204.2 nm ± 6.2 nm) (Fig. 2G).

Our data supports a model in which the MPS does not extend continuously into the distal outgrowing axon. Instead, the MPS assembles from multiple “MPS seeds” that grow and coalesce into a single scaffold. The formation of the MPS in an outgrowing axon can be divided into an assembly phase with multilocular periodic spectrin patches developing along the axon, an elongation phase where single patches extend their size and the number of patches further increases, and a final coalescence phase where the MPS is closed (Fig. 3).

### Spectrin periodicity is increased early after regeneration

**Figure 4:**
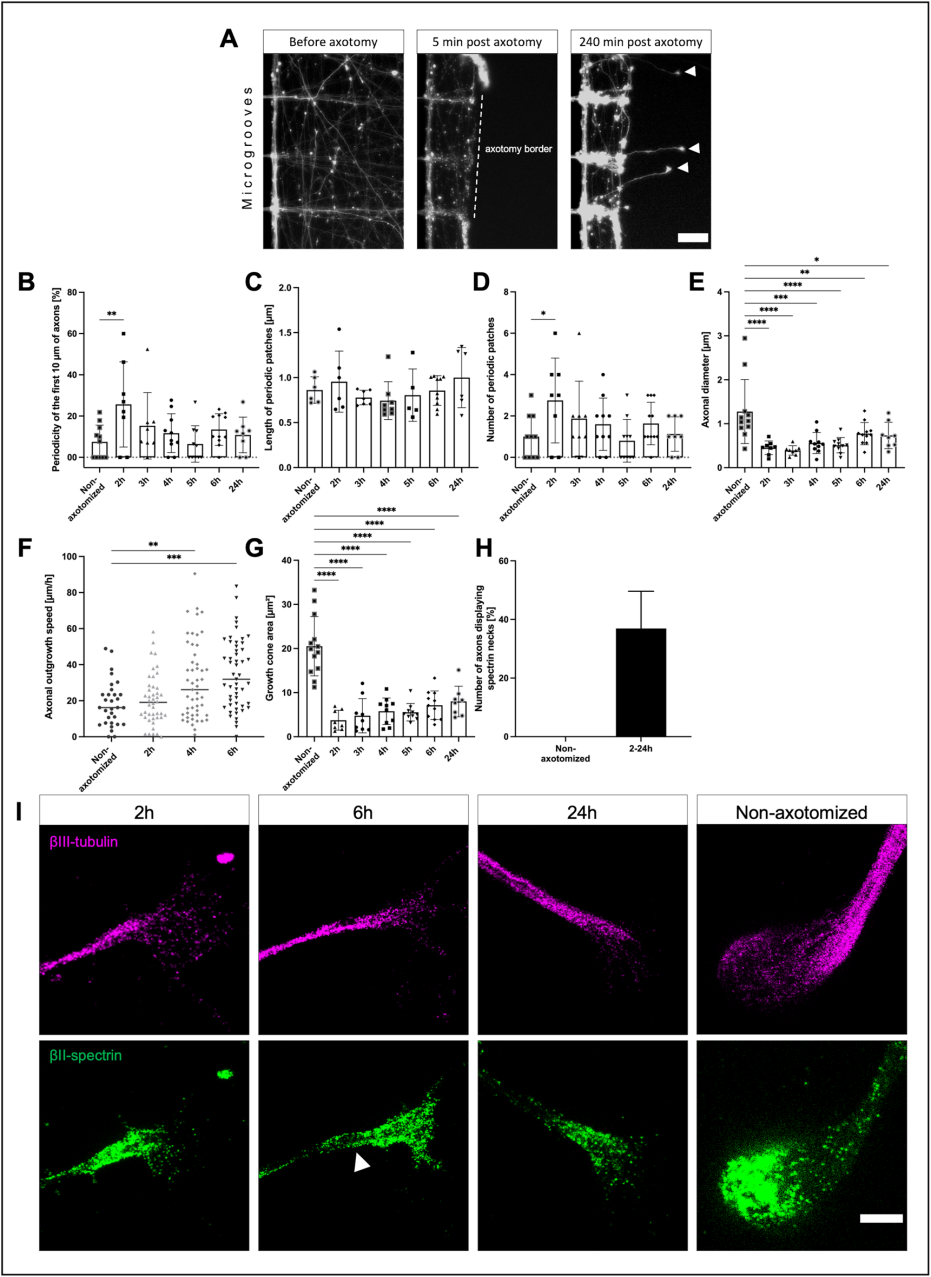
Comparison of growth cone morphology and βII-spectrin periodicity of the first 10 µm of axon proximal to the growth cone in samples of non-axotomized and regenerating neurons. (**A**) Graphical explanation of the method used for marking regenerating axons. Microscopic images of the same section of the axonal compartment were acquired before axotomy, five minutes after, and 2–24 h after axotomy. For detailed information, see the methods section. White arrowheads indicate regenerating axons. Scale bar is 30 µm. **(B**) Comparison of periodicity in the first 10 µm of the axon, starting from the growth cone base. Data of different time points after axotomy and non-axotomized axons are shown. The 2 h time point shows a significant increase in periodicity. Bars represent mean ± SD. One-way ANOVA, followed by Dunnett’s multiple comparisons test, was performed. At least 8 axons were analyzed per condition, n = 3, 67 axons were analyzed in total. (**C** and **D**) Comparison of the average length and number of periodic patches in the first 10 µm of axon adjacent to the growth cone. Data of different time points after axotomy and non-axotomized axons are shown. No significant differences were detected for periodic patch length (**C**). The average number of periodic patches was significantly increased at the 2 h time point, compared to non-axotomized axons (**D**). One-way ANOVA was performed, followed by Dunnett’s multiple comparisons test. Bars represent mean ± SD. At least 8 axons were analyzed per condition, n = 3, 67 axons were analyzed in total. (**E**) Comparison of axonal diameter in the first 10 µm of the axon, starting from the growth cone base. Data of different time points after axotomy and non-axotomized axons are shown. The reduction of the axonal diameter of the different time points after axotomy is significant, compared to non-axotomized axons, whereas only the 2 h time point shows a significant increase in periodicity (**B**). Bars represent mean ± SD, one-way ANOVA, followed by Dunnett’s multiple comparisons test was performed. At least 8 axons were analyzed per condition, n = 3, 67 axons were analyzed in total. (**F**) Comparison of axonal outgrowth speed in cortical neurons fixed at DIV 10. The 4 h and 6 h time points showed significantly increased outgrowth speed compared to non-axotomized axons. The line represents mean, one-way ANOVA, followed by Dunnett’s multiple comparisons test was performed. At least 31 Axons were analyzed per condition, n = 3, 177 axons were analyzed in total. (**G**), Comparison of growth cone area of different time points after axotomy and non-axotomized axons, quantified in spectrin-stained axons. The growth cone area of regenerating axons is significantly reduced compared to non-axotomized axons. Bars represent mean ± SD; one-way ANOVA, followed by Dunnett’s multiple comparisons test, was performed. At least 8 axons were analyzed per condition, n = 3, 67 axons were analyzed in total. (**H**) Comparison of regenerating axons of combined time points and non-axotomized axons displaying spectrin necks. Non-axotomized axons did not display spectrin necks, whereas regenerating axons showed variating percentages of spectrin necks. A spectrin neck was defined as an at least 600 nm long increase of spectrin signal at the axonal segment directly connected to the growth cone, not longer than 3 µm. At least 8 axons were analyzed per condition, n = 3, 67 axons were analyzed in total. (**I**) Representative STED images of growth cone sizes at different time points after axotomy and non-axotomized. Notice the size difference between non-axotomized and regenerating axons. White arrowhead points to spectrin neck. The scale bar is 2 µm.

Our next set of experiments aimed to analyze the morphological and cytoskeletal changes in regenerating axons following an axotomy. We performed axotomy by vacuum aspiration in microfluidic chambers and fixed the neurons and regenerating axons at different time points (2, 3, 4, 5, 6, and 24 h) after axotomy. We then analyzed different morphological features of the GC and the axon and analyzed the development of the MPS in the axon. Because of the different lengths of the regenerating axons at different time points, we compared periodicity values only at the first 10 µm close to the GC at all time points. Each analyzed axon was traced over time to confirm its axotomy and to reliably exclude the few non-axotomized axons growing through the microgrooves from the analysis (Fig. 4A; for further details, see methods section). The non-axotomized group comprised native axons before axotomy. Regeneration was first noticed at 2 h after axotomy. Axons extended in length over the examined period.

Surprisingly, regenerating axons developed their MPS much faster than the non-axotomized axons with a significant, almost 3.5-fold higher spectrin periodicity in the first 10 µm at 2 h after axotomy in regenerating axons compared to non-axotomized axons (Fig. 4B) (25.7% ± 7.3% vs. 7.5% ± 2.4%).

As Fig. 4C shows**,** the length of periodic patches was not significantly increased in regenerating axons, compared to non-axotomized axons (0.96 µm ± 0.1 µm vs. 0.86 µm ± 0.06 µm). However, the number of periodic patches was increased almost three-fold at the 2-h time point, compared to non-axotomized axons (2.7 ± 0.7 vs. 1.0 ± 0.3; Fig. 4D).

We thus conclude that after axotomy, the axons transiently form more periodic spectrin patches in the outgrowing part of the axon resulting in a higher degree of periodicity and a quicker assembly phase compared to non-axotomized axons. Interestingly, at later time points after axotomy, the lesioned axons then resembled more and more the characteristics of the non-axotomized axons with regards to their periodicity and number of periodic patches.

The axonal diameter of the segment adjacent to the GC was decreased in regenerating axons compared to non-axotomized axons (Fig. 4E). At 2 h after axotomy, the axonal diameter was reduced almost three-fold (2 h: 0.5 µm ± 0.1 µm vs. non-axotomized: 1.3 µm ± 0.2 µm). The axonal diameter increased in the following hours but was still significantly reduced almost two-fold up to 24 h after the axotomy (24 h: 0.7 µm ± 0.1 µm).

To test whether the outgrowth speed of regenerating axons was faster or not, we performed time-lapse microscopy and measured the outgrowth velocity of the GC in the time-lapse video. As Fig. 4F shows, we saw a significant increase in outgrowth velocity 4 and 6 h after axotomy but not 2 h after axotomy (non-axotomized: 18.3 µm/h ± 2.3 µm/h, 2 h: 20.5 µm/h ± 2.1 µm/h, 4 h: 30.8 µm/h ± 3.0 µm/h, and 6 h after axotomy: 34.5 µm/h ± 2.7 µm/h). Thus, the outgrowth velocity is not correlated to the higher spectrin periodicity.

To analyze whether the newly formed GC after axotomy differed morphologically from the non-axotomized one, we measured the area of non-axotomized and regenerating GCs at different time points. As Fig. 4G shows, the area of regenerating GCs at 2 h after axotomy was reduced five-fold, compared to non-axotomized axons. The GC area increased by two times over 24 h but did not reach the size of non-axotomized axons in this timespan (2 h: 3.7 µm^2^ ± 0.8 µm^2^, 24 h: 8.0 µm^2^ ± 1.2 µm^2^, and non-axotomized axons: 20.5 µm^2^ ± 1.9 µm^2^). We observed a similar reduction regarding the length and width of the GCs; for detailed information, see Supplemental Fig. 4.

In order to test whether regenerating GCs also show an enrichment of spectrin, we performed line intensity scans along the GC and axon, similar to the non-axotomized axons. To compare the different time points, we averaged the intensity along the length of the GC and compared it to the intensity along the first 10 µm of axon next to the GC. The intensity of tubulin was decreased in the GC throughout all time points after axotomy, as seen before in the non-axotomized axons, probably due to the splaying of microtubules in the GC (see Supplemental Fig. 5). Interestingly, though a trend could be assumed, the enrichment of spectrin inside the GC was not persistent in regenerating GCs, except for the 6 h time point (non-axotomized axons: GC 0.51 vs. axon 0.33, 2 h: GC 0.4 vs. axon 0.28, 4 h: GC 0.52 vs. axon 0.34, 6 h: GC 0.56 vs. axon 0.33, 24 h: GC 0.6 vs. axon 0.49).

Some axons showed an enrichment of spectrin close to the GC, which we will refer to as “spectrin neck”. A spectrin neck was defined as an at least 600 nm long increase of spectrin signal, relative to the more proximal axon, at the axonal segment directly connected to the GC, not longer than 3 µm. The first 4 µm of axon adjacent to the GC were measured using line intensity scans. The increase had to be higher than the 75th percentile of the measured 4 µm, and the first micrometer had to have 1.5 times higher intensity values than the 2nd–4th micrometer. A fraction of 36.9% of regenerating axons showed spectrin necks compared to none of the non-axotomized axons in our sample size (Fig. 4H,I). See Supplemental Fig. 6 for combined line intensity scans of axons with or without spectrin necks.

To sum up the results of the axotomy experiments, regenerating axons compared to non-axotomized axons exhibit a transiently higher degree of spectrin periodicity based on an increased periodic spectrin patch density, a persistent smaller axon diameter, and a reduced growth cone size. Outgrowth velocity is increased at 4 and 6 h after axotomy in the regenerating axons. Some regenerating axons show an accumulation of spectrin at the base of the GC.

### Inhibition of calpain increased periodicity and the number of regenerating axons shortly after axotomy

**Figure 5:**
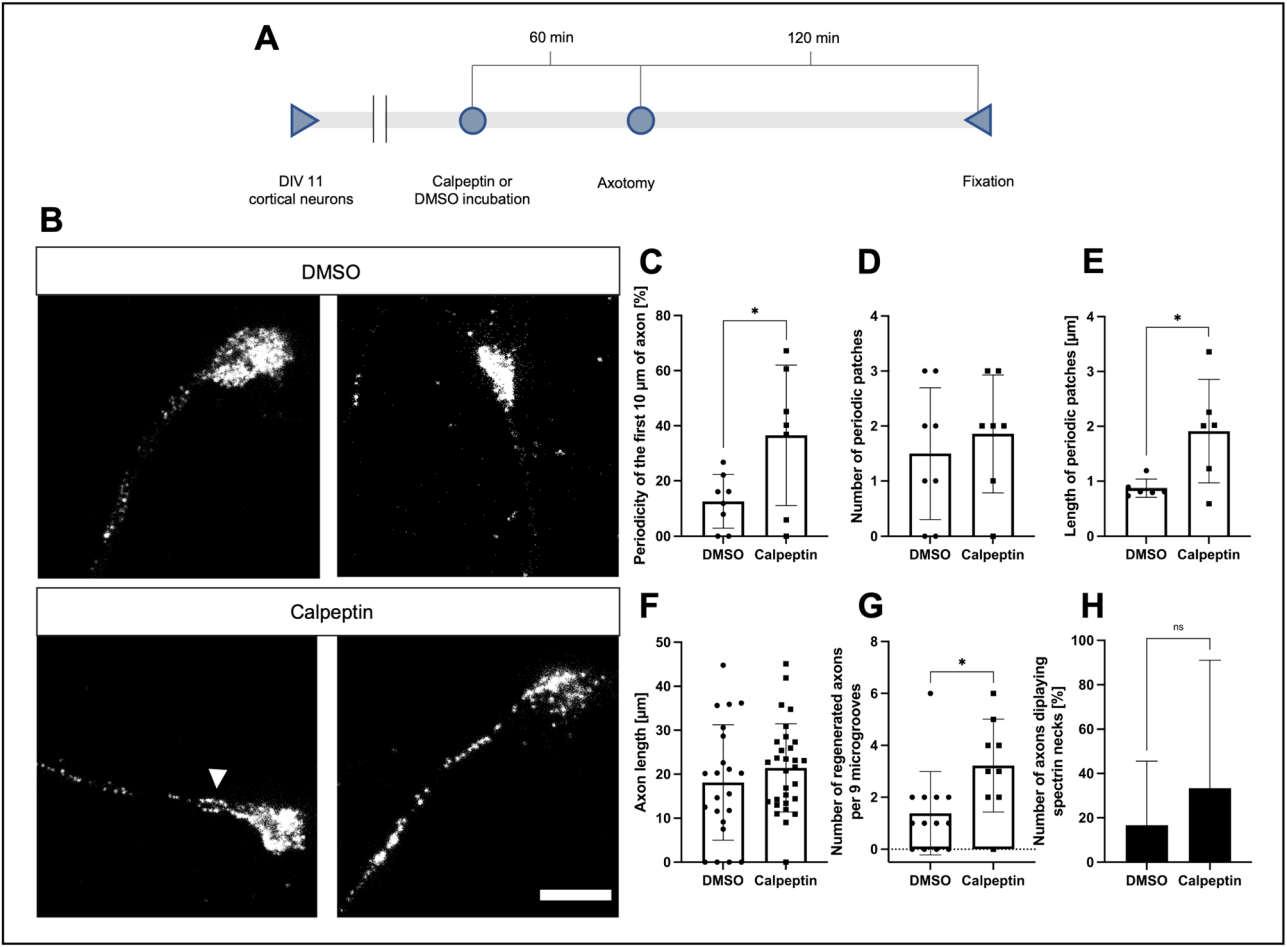
Effects of calpeptin treatment on spectrin periodicity and regeneration of axons after axotomy. **(A**) Schematic display of used test setup. 10 µM of calpeptin or equal amounts of DMSO were used for incubation. STED imaging was performed following fixation. (**B**) Representative STED images of cortical neurons treated with calpeptin or DMSO before axotomy, fixation after 2 h, scale bar 2 µm. The arrowhead points to a spectrin neck at the base of the growth cone. (**C–E**) Quantification of parameters for MPS development along the axon in regenerating axons treated with calpeptin, compared to regenerating DMSO controls. Analyzed were the first 10 µm of axon close to the growth cone. Periodicity (**C**) and size of periodic patches (**E**) were increased in calpeptin-treated axons. Bars represent mean ± SD. Unpaired t test (**C** and **D**) or Kolmogorov–Smirnov test (**E**) was performed. At least 7 axons were analyzed per condition, n = 3, 15 axons were analyzed in total. (**F** and **G**) Graphical display of axon length of regenerating neurons 2 h after axotomy. (**F**) shows no significant increase in axon length, while (**G**) shows a significant increase in regenerating axons per 9 microgrooves. Bars represent mean ± SD. Non-parametric Mann–Whitney U test was performed. At least 22 axons were analyzed per condition, n = 3, 52 axons were analyzed in total. (**G**) at least 27 microgrooves were analyzed per condition, n = 3, 198 microgrooves were analyzed in total. (**H**) Quantification of axons, treated with DMSO or calpeptin, displaying spectrin necks. Calpeptin-treated axons displayed a higher percentage of growth cones with spectrin necks. A spectrin neck was defined as an at least 600 nm long increase of spectrin signal at the axonal segment directly connected to the growth cone, not longer than 3 µm. At least 7 axons were analyzed per condition, n = 3, 15 axons were analyzed in total.

Since spectrin periodicity was increased early in regenerating axons, we wondered how inhibition of calpain might influence the growth behavior of axons. To inhibit calpain and putatively further increase periodicity after axotomy, we treated the neurons with the calpain inhibitor calpeptin (10 µM dissolved in dimethylsulfoxide (DMSO) or DMSO alone as control, in both conditions, DMSO was diluted to 0.012% in media), performed an axotomy, and analyzed the axons 2 h after axotomy (Fig. 5A,B). Calpain is a spectrin cleavage enzyme that is activated early after axotomy leading to a destruction of spectrin structures^22^, among other substrates.

The calpeptin treatment led to a more than threefold increase in spectrin periodicity at 2 h after axotomy in the treated axons compared to control (36.6% ± 9.6% vs. 12.7% ± 3.4%) (Fig. 5C). Interestingly, the axonal diameter did not differ between both groups (Supplemental Fig. 7).

The number of periodic spectrin patches was not significantly changed in calpeptin-treated neurons compared to control (1.9 ± 0.4 vs. 1.5 ± 0.4) (Fig. 5D). The length of the periodic spectrin patches, however, was increased by 2.5-times in calpeptin treated neurons, compared to DMSO treated neurons (1.6 µm ± 0.4 µm vs. 0.7 µm ± 0.2 µm) (Fig. 5E). The mean number of periodic patches seems decreased in both conditions compared to the previous experiment at 2 h, but this difference was not statistically significant.

The length of the regenerating segment of the axons was not significantly different between calpeptin or DMSO treated neurons (21.4 µm ± 1.8 µm vs. 18.1 µm vs. 2.8 µm) (Fig. 5F). Interestingly, the number of regenerating axons per 9 microgrooves (9 microgrooves are in the viewfinder at 20 × magnification) was significantly higher in analyzed images of calpeptin-treated chambers, compared to DMSO control group (3.2 ± 0.6 vs. 1.4 ± 0.4) (Fig. 5G). Likewise to the prior experiments, we also analyzed the distribution of spectrin along the GC and the distal axon using line intensity scans. Interestingly, calpeptin treated axons 2 h after axotomy showed a significant enrichment of spectrin inside the GC, but not the DMSO treated group, nor the non-treated group of the previous experiment at 2 h after axotomy (DMSO: GC 0.25 vs. axon 0.25, calpeptin: GC 0.41 vs. axon 0.25; see Supplemental Fig. 8). Axons treated with calpeptin did not show spectrin necks significantly more often than in the control group (33.3% vs. 16.7%) (Fig. 5H).

Inhibition of calpain thus significantly enhances the elongation phase of MPS formation, leading to increased spectrin periodicity without influencing the assembly phase. This does not increase outgrowth velocity but enables more axons to regenerate within the analyzed time period.

## Discussion

In this study, we analyzed the formation and development of the MPS in the outgrowing axon. We report that the developing MPS is possibly organized in periodic patches that grow in size and number and coalesce over time during its maturation. We provide further evidence that the MPS develops in a proximal to distal pattern. We also show that regenerating axons after axotomy acquire periodicity faster over a transient timespan.

We found an enrichment of spectrin inside the axonal GC in line with previous studies^23–26^. Elevated spectrin levels in the GC could resemble a pool of spectrin tetramers that are later incorporated into the growing axon. This enrichment was present in non-axotomized GCs but was not significant in regenerating GCs after axotomy, except for one time point. Therefore, regenerating GCs may fail to re-establish spectrin levels prior to axotomy. This could be due to a higher demand for spectrin molecules during regeneration.

The MPS has been found along the axon, in dendrites, the AIS, and the neck of dendritic spines^27^ and in the soma^15^. In outgrowing axons, the MPS has been suggested to develop in a proximal to distal pattern^12–14^. We can specify that growing axons display a characteristic spatial and temporal decrease of spectrin periodicity in a gradient from the proximal axonal regions towards the distal growth cone. We show that the MPS does not extend in a continuous pattern along the axon. Instead, multiple periodic patches are assembled along the growing axon. This assembly happens close to the GC, at a frequency of about one patch per 10 µm. These periodic patches double in number over a timespan of about 11 h, then increase in size and conflate as maturation continues to form the mature MPS finally. This maturation happens gradually as the axon grows, resulting in a gradient from mature MPS in the mid-axon towards immature MPS at the GC base. Our data, however, has to be interpreted with caution, as we infer dynamic properties of the MPS from fixed cells.

In neural stem cells, short periodic patches of a 1D arrangement of actin and spectrin were reported in the soma^15^. Matured neurons then displayed a developed MPS. We believe that our finding of periodic patches in the distal axon shows the same or a similar process of MPS development. Interestingly, we show both conditions (periodic patches and developed MPS) in the same axon, with the difference between the conditions being the distance to the soma.

We suggest the GC or its neck as a possible assembly site of periodic patches for three reasons: First, the GC sits on top of the growing axon where spectrin assembly naturally starts. Second, our data shows periodic patches in the axonal segment adjacent to the GC. Third, spectrin is highly enriched within the GC. It could therefore function as a pool for the implementation of spectrin into the growing axon. Interestingly, the number of periodic patches also increases at distances of 100–200 µm from the GC. This increase could be explained by an assembly machinery that stays on the way as the axon extends after integration into the axon at the GC. Zhong et al. demonstrated that formation of the MPS in dendrites is dependent on the local concentration of spectrin and the presence of ankyrin B^12^. Surprisingly, we did not detect periodic patches or periodicity inside the GC, despite higher concentrations of spectrin and ankyrin B^23^. This points to other assembly factors that might not be present in the GC but in the axonal segment close to the GC. Our findings, therefore, shine a new light on the role of the GC and its neck in the formation of the axonal MPS.

A role of the MPS in maintaining axonal diameter has been described in previous studies^7,11^. We show here that the axonal diameter decreases while the MPS is formed. The finding that the youngest part of the axon shows the lowest periodicity, but greatest diameter, supports the idea of a progressive constriction of the axonal diameter by the MPS^11^. It has also been shown that the diameter of actin rings decreased over time in culture^11^. Furthermore, knockout of α-adducin, also distributed periodically along the axon, increased the axonal diameter, without changes in the spacing of actin rings^11^. Previous studies found recruitment of adducin into the MPS after DIV 8^12^. It is also well known that non-muscle myosin II (NMII) has a role in regulating axonal diameter^28^ and is distributed throughout the MPS^7,8,29^. We found that calpain inhibition did not affect the axonal diameter in young, regenerating axons. Interestingly, degeneration of the axon and its MPS through trophic factor withdrawal did also not lead to an increase in axonal diameter^16^.

The role of the MPS in axonal degeneration has been studied previously^16,17^. However, the MPS has not been studied in regenerating axons so far. Neuronal regeneration is an important process that needs to be activated in many neurological conditions in order to compensate for the disease-associated damage of nerve tissue, e.g., in spinal cord injury, stroke, or neurodegenerative diseases^30^.

We show here that axon morphology and the formation of the MPS in regenerating axons differ significantly from non-axotomized axon outgrowth. Most strikingly, the axon diameter and growth cone size were significantly reduced in regenerating axons compared to non-axotomized axons at all examined time points within 24 h after axotomy. This reduction correlated with an increased outgrowth speed of the regenerating axons at 4 and 6 h after axotomy.

At 2 h after axotomy, spectrin periodicity and the number of spectrin patches were significantly increased in the newly formed axon but reached “normal” values at later time points. This suggests that the assembly phase and most likely also the coalescence phase of MPS formation are enhanced early after axotomy. Since we accurately checked that all analyzed axons had been axotomized and evaluated only the newly formed part of the axons, we are confident that the observed alterations of spectrin MPS formation are characteristic of the regenerating axon and not confounded by residual proximal parts of the axotomized axon. A plausible explanation for the observations is that the molecular machinery necessary for assembly and coalescence of spectrin patches is already in place in the more proximal part of the axon that remains after axotomy and can thus more quickly be employed for MPS formation. The decrease (i.e., normalization) of the number of periodic patches at later time points could be due to the increased outgrowth speed in the following hours, which would dilute a putatively still increased number of patches.

Also, spectrin itself is more abundant in the distal regenerating axon as compared to non-axotomized axons. In around 25% of all regenerating axons, we measured an accumulation of spectrin adjacent to the GC, which we termed spectrin neck. Spectrin necks were not observed in non-axotomized axons in our sample size and could therefore be specific for regenerating axons. The underlying mechanism for spectrin neck development might be increased local translation of spectrin RNA^31^, altered axonal transport^13^, or local cleavage with increased spectrin reutilization^17^.

Although hypothesized in a review by Leite and Sousa^32^, our finding of increased spectrin levels and periodicity in regenerating axons is particularly surprising, considering the degradation of the MPS in degenerating axons following axotomy or NGF-withdrawal, presented in previous studies^16,17^. A possible explanation could be increased levels of free spectrin molecules after the axotomy. It has been shown that overexpression of spectrin increases periodicity in dendrites^12^. Since only a fraction of the axotomized axons regenerates while another fraction degenerates and only the regenerating axons were selected in our analysis, it can also be speculated that the observed features of the cytoskeleton are structural prerequisites that enable lesioned axons to regenerate after a lesion quickly.

It is well known that axotomy leads to a rapid calcium influx into the axoplasm and activation of the calcium-dependent protease calpain that induces the consecutive destruction of the axonal cytoskeleton^33,34^. Inhibition of calpain, e.g., via calpeptin, has been shown to inhibit axonal degeneration after lesion, at least within the first hours after axotomy^22,33^. We thus analyzed the effects of calpeptin treatment on the spectrin cytoskeleton on the nanoscale. Calpeptin treatment indeed led to a significant increase in spectrin periodicity in regenerating axons. Interestingly, upon calpeptin treatment, the size of the periodic patches was increased, but not their number. Thus, calpain inhibition may not interfere with spectrin assembly but enhances the elongation of periodic spectrin patches. A possible explanation for this finding is a decreased degradation of spectrin molecules in the distal axon caused by calpain inhibition, which could possibly raise the concentration of spectrin in the GC. This is supported by our finding of increased spectrin levels in the GC in calpeptin treated axons. As shown before by Zhong et al. the formation of the MPS in dendrites is dependent on the local concentration of spectrin and the presence of ankyrin B^12^.

The increased and more rapidly evolving spectrin periodicity did not translate into an increased length of the regenerating axons. This is in line with previous studies that reported adverse effects of calpeptin on growth cone stability and axonal regeneration^35^. It is proposed that a constant turnover of the cytoskeletal molecules is essential for the highly dynamic growth cone and axonal elongation. However, we found an increased number of regenerating axons in the calpeptin-treated neurons. Thus, enhanced MPS-formation might set a basis for more axonal regeneration.

Due to the time-consuming semi-manual nanoscale analysis of the axonal cytoskeleton, we were only able to include a relatively small sample size. As previously demonstrated, this could be improved by adopting automated unbiased sampling and quantitative analysis^16,36^. As dynamic properties of the MPS are inferred from fixed cells, live-STED microscopy should be performed to further analyze the formation of the MPS in periodic spectrin patches. This, however, was not possible in our lab setup.

Taken together, we demonstrate here that the MPS is formed in the region 250 µm proximal from the GC in three possible phases: assembly of periodic spectrin patches adjacent to the GC, subsequent elongation of periodic patches, and finally coalescence of periodic patches into a mature MPS. After a lesion, the outgrowth of regenerating axons is characterized by a greater speed, thinner axon diameter, smaller growth cones, and at least transiently an increased number of periodic patches and higher periodicity. Inhibition of the spectrin-severing protease calpain increased spectrin periodicity and enhanced the elongation phase in regenerating axons. Future research should explore the underlying molecular mechanisms and test different substances that specifically alter MPS dynamics.

## Materials and methods

### Cell culture, viral transduction, and axotomy

The use of E18 Wistar rat embryos for isolation of cortical tissue was conducted according to the approved experimental animal licenses (33.9-42502-04-11/0408) issued by the responsible animal welfare authority (Niedersächsisches Landesamt für Verbraucherschutz und Lebensmittelsicherheit) and controlled by the local animal welfare committee of the University Medical Center Göttingen, Germany. All animal experiments were performed according to the ARRIVE guidelines.

Primary cortical neurons were isolated from E18 Wistar rat embryos as described before^37^. In short, the dissected tissue was incubated with 1 ml trypsin (Sigma Aldrich, St. Louis, MO, USA; #T9935, 25.000 U/ml) for 15 min at 37 °C. Approximately 1 min before the trypsinization was complete, 50 µl of DNase I (Roche, Basel, Swiss; #11284932001, 2000 U/mg) were added. The suspension was centrifuged shortly, and the supernatant was removed. Pellet was then resuspended in 1 ml fetal bovine serum (FBS; Biochrom, Berlin, Germany) and carefully triturated. If tissue clumps were still present, more DNase was supplemented. The supernatant was then transferred into a 15 ml falcon tube, and the remaining tissue was again triturated in 1 ml of media. The supernatant was transferred again, and the falcon tube was centrifuged. Neurons were resuspended in 500 µl Neurobasal medium (Gibco, Waltham, MA, USA), supplemented with 10% of FBS, 2% of 50 × B27 Supplement (Gibco), 1% Penicillin-Streptomycin-Neomycin (Gibco), 0.5% holo-Transferrin human (Applichem, Darmstadt, Germany), and 0.25% L-glutamax (Gibco). Neurons were seeded at a density of 60,000 cells per chamber into the somatic compartment of a microfluidic chamber (MFC) (XONA microfluidics, Research Triangle Park, NC, USA). MFCs were mounted onto 35 mm FluoroDishes (World Precision Instruments, Inc, Sarasota, FL, USA), precoated with 0.1 mg/ml poly-L-ornithine (Sigma Aldrich, P3655) in borate buffer solution (Sigma Aldrich, P8638), and 0.1% laminin (Sigma Aldrich, L2020) as described previously^22^.

All cell cultures used for STED microscopy were transduced with an AAV 1/2, expressing EGFP (GenBank HQ416702.1), kindly gifted by Uwe Michel, Göttingen, as described before^38^. The stock solution had a titer of 5.6 × 10^7^ transduction units (TU). Expression was driven by a human synapsin-1 promoter and a human h1 promoter. The virus was employed with a titer of 0.5 × 10^7^ TU per chamber and added to the medium on DIV 4. Usually, a transduction rate of > 90% was achieved, with no significant toxicity. The medium was augmented from DIV 4–11 with 10 ng/µl of ciliary neurotrophic factor (CNTF, Pepro Tech, Rocky Hill, NJ, USA) and brain-derived neurotrophic factor (BDNF, Pepro-Tech). 10% of FBS was added to the medium in the first week but was omitted after DIV 6 to prevent the overpopulation of astrocytes. Cultured neurons were maintained with frequent medium exchange (every 2 to 3 days) and kept at 37 °C and 5% CO_2_. Cells looked healthy, and no signs of apoptosis were observed until DIV 11 following this protocol.

Axotomy was performed on DIV 11 by vacuum aspiration of the media in the axonal compartment as described elsewhere^18^. In short, the medium was aspirated using a cell culture pump (Schütt Labortechnik, Göttingen, Germany) until an air bubble passed through the axonal compartment, dissecting the axons. This procedure was repeated 2–4 times, and successful axotomy was assessed using a standard cell culture microscope (Zeiss, Jena, Germany). For treatment of neurons with calpeptin (Sigma Aldrich, #C8999), axons were incubated with 10 µM of calpeptin in DMSO (Applichem) or equal amounts of DMSO for 1 h prior to the axotomy, administered only into the axonal compartment.

### Immunocytochemistry

All neuronal cultures were fixed on DIV 11. Axotomized neurons were fixed either 2, 3, 4, 5, 6, or 24 h post-axotomy. Cells were washed with PBS (Applichem) and fixed with pure methanol (Applichem) for 10 min at − 20 °C. Afterward, fixed cells were washed three times with PBS and stored at 4 °C in PBS if not immediately processed. Neuronal cultures used for staining actin were fixed for 1 h using 4% paraformaldehyde (PFA; Applichem) in PHEM buffer, with 0.5% glutaraldehyde (Applichem), adapted from^39^.

For immunocytochemistry (ICC), MFCs were washed with PBS. If neurons were fixed with PFA, quenching with glycine (Applichem) and ammonium chloride (Applichem; both 100 mM in PBS) was performed to eliminate free aldehyde groups. After quenching (in case of PFA fixation) or initial washing (upon methanol fixation), cells were permeabilized with Triton X100 (0.3% in PBS; Applichem) for 5 min at room temperature (RT), MFCs were then incubated in blocking solution containing 2% bovine serum albumin (BSA) in PBS for 20 min at RT (Jackson Immuno Research, West Grove, PA, USA, #001-000-162). The blocking solution was removed, and primary antibodies were added for 1 h at RT.

After the primary antibody reaction, suitable secondary antibodies or phalloidin STAR RED were added. Incubation time was set to 45 min and was performed at 37 °C. If not immediately imaged, stained neurons were stored light-protected in PBS at 4 °C.

Monoclonal primary antibodies directed against βII-spectrin were obtained from BD (BD Bioscience, Franklin Lakes, NJ, USA; mouse, #612563, 1:200), and rabbit βIII-tubulin antibodies were from Cell Signaling (Cambridge, UK; #5568S, 1:100). Polyclonal secondary antibodies goat anti-mouse Alexa594 were obtained from Invitrogen (Waltham, MA, USA; #A-11005, 1:100), and goat anti-rabbit STAR635p was from Abberior (#2-0012-007-2, 1:100, Göttingen, Germany) as was Phalloidin-STAR RED (#2-0205-011-7, 1:100). Calpeptin was obtained from Sigma (#C8999, 10 µM).

### Identification of regenerated axons

To identify and mark regenerated axons for STED microscopy, fluorescence live microscopy of neuronal cultures was performed. Cultures were imaged using an inverted fluorescence microscope (Axio Observer.Z1, Zeiss), equipped with an incubation chamber (Incubator PM 2000 RBT, PeCon, Erbach an der Donau, Germany), set to 37 °C and 5% CO_2_. MFCs were placed inside the incubation chamber using a custom-made, 3D-printed template to seal the chamber against loss of CO_2_. Images were acquired at 20 × magnification before axotomy, directly after, and at respective time points during regeneration (2, 3, 4, 5, 6, and 24 h after axotomy). Using the “multiview” function of the Zen 2.5 software (blue edition, Zeiss), images were arranged side by side and aligned. After confirmation of a sufficient axotomy, the regenerating GCs that were not present before or shortly after the axotomy were marked and sorted out. Segments of the MFCs, showing neuronal somata on the axonal side were excluded. The microgrooves were numbered, and STED microscopy was performed only with axons previously identified as regenerating axons.

### Analysis of axonal outgrowth speed

Axonal outgrowth speed was assessed using time-lapse microscopy. In short, cells were cultured as described above. Several 10-min-long time-lapse videos of the axonal compartment were captured before axotomy and 2, 4, and 6 h after axotomy, at 40 × magnification, using the setup described above. The position of the GC was marked in the first and last image of the video, and the distance traveled by the GC was then measured using the open-source freeware Fiji^40^.

### Stimulated emission depletion (STED) microscopy

STED microscopy was performed to analyze MPS development in regenerating and non-axotomized axons. At least two regenerated axons were recorded per condition and chamber, starting at the GC and moving retrogradely towards the axotomy border. At 24 h post-axotomy, axons were too long to be followed back to the axotomy border. Therefore, at least three images were acquired along the axon, starting from the GC. Non-axotomized axons were imaged likewise. Two-color STED microscopy was performed using a quad scanning STED microscope (Abberior Instruments, Göttingen, Germany) equipped with an UPlanSApo 100×/1,40 Oil objective (Olympus, Tokyo, Japan). An IX83 Olympus microscope with 4-color LED illumination source and a monochrome widefield camera with a 1/2” CCD chip and 1280 × 960 pixels were used as a platform. Pixel size was set to 20–25 nm with a pinhole size of 0.7 airy units. Dwell times were set to 10 µs. Lasers with wavelengths of 561 nm and 640 nm were used for excitation of Alexa 594 or STAR 635p/STAR RED, respectively. Depletion was performed using a 775 nm pulsed STED laser. Images were acquired using the Imspector software (Abberior, Göttingen, Germany)^41^.

### Analysis of STED images

The brightness and contrast of the obtained STED images were adjusted using a custom-made macro and Fiji, mimicking the “auto-function” of the brightness and contrast tool, and converted into an 8-bit tagged image file format. If necessary, images of axon segments were stitched manually with MosaicJ^42^ upon deactivated smart color conversion, rotation, and blending. Images of the different cytoskeletal components were analyzed independently using Fiji. As for the evaluation of GC morphology (length, width, and area), the margin was outlined by applying the “polygon selection” tool. The length was determined by drawing a straight line from the GC base to the farthest point of the previously constructed polygon. The width was set to the longest straight within the polygon, being orthogonal to the length-line. A spline-fitted segmented line, with a thickness covering the axons width at the narrowest part, was drawn from the tip of the GC along the axon towards the axotomy border to plot intensity curves. To reduce data size and avoid outliers, we summarized the measurements for every 1 µm and normalized each dataset to its maximum. The axon itself was further evaluated regarding diameter and βII-spectrin periodicity in 10 µm segments, using the self-programmed “Segmenter” macro for the segmentation of nonlinear structures. The diameter was measured along the axon, starting at the GC neck, for every 10 µm. We calculated βII-spectrin periodicity by measuring every periodic patch in a segment and dividing the total length by 10 µm. The number of periodic patches per segment and the average patch size were also assessed. One periodic patch was defined as at least three individual βII-spectrin-signals inside the axon with a mean distance of 150–250 nm between spectrin signals. This was assessed by performing line intensity scans along periodic patches, confirming the periodic distance of intensity peaks. Primary axons were analyzed likewise, but due to the absence of an axotomy border, the first 250 µm of the axon, starting at the GC base, were considered. To analyze the average spacing of spectrin tetramers, line intensity scans were performed in spectrin stains along periodic patches at distances of 0, 100, and 200 µm from the GC in all non-axotomized axons. The distances between periodic signal peaks of spectrin were then measured. In co-stainings of actin and spectrin (Fig. 1E and Supplemental Fig. 3), actin is pseudo-colored using the CET-L15 colormap by Peter Kovesi^43^ (colorcet.com).

### Statistics

Statistical evaluation was performed using GraphPad PRISM 9.1 (GraphPad Software, Inc, San Diego, CA, USA). Two groups were compared using unpaired Student’s t test (two-tailed). Non-parametric Mann–Whitney U or Kolmogorov–Smirnov tests were used if the assumptions for the t test were not met. Ordinary one-way ANOVA was conducted to compare two or more groups, followed by Dunnett’s multiple comparisons test. In general, mean values ± SEM are described unless otherwise noted, and at least three independent experiments were analyzed. Wherever possible, individual values were displayed in graphs. Significant differences between compared groups are exhibited as follows: *p < 0.05, **p < 0.01, ***p < 0.001, ****p < 0.0001.

## Supplementary Information

Supplementary Information.

## Data Availability

All data generated or analyzed during this study are included in this published article (and its supplementary information files). Original micrographs are available from the corresponding author upon reasonable request.
