# Supplementary material for: Cytoskeletal assembly in axonal outgrowth and regeneration analyzed on the nanoscale
Source: Sci Rep. 2022 Aug 23;12:14387. doi: 10.1038/s41598-022-18562-5 (PMC9399097; doi:10.1038/s41598-022-18562-5)
Supplement: Supplementary file 1 — Supplementary Information. [file 41598_2022_18562_MOESM1_ESM.docx]

## Supplemental Figures


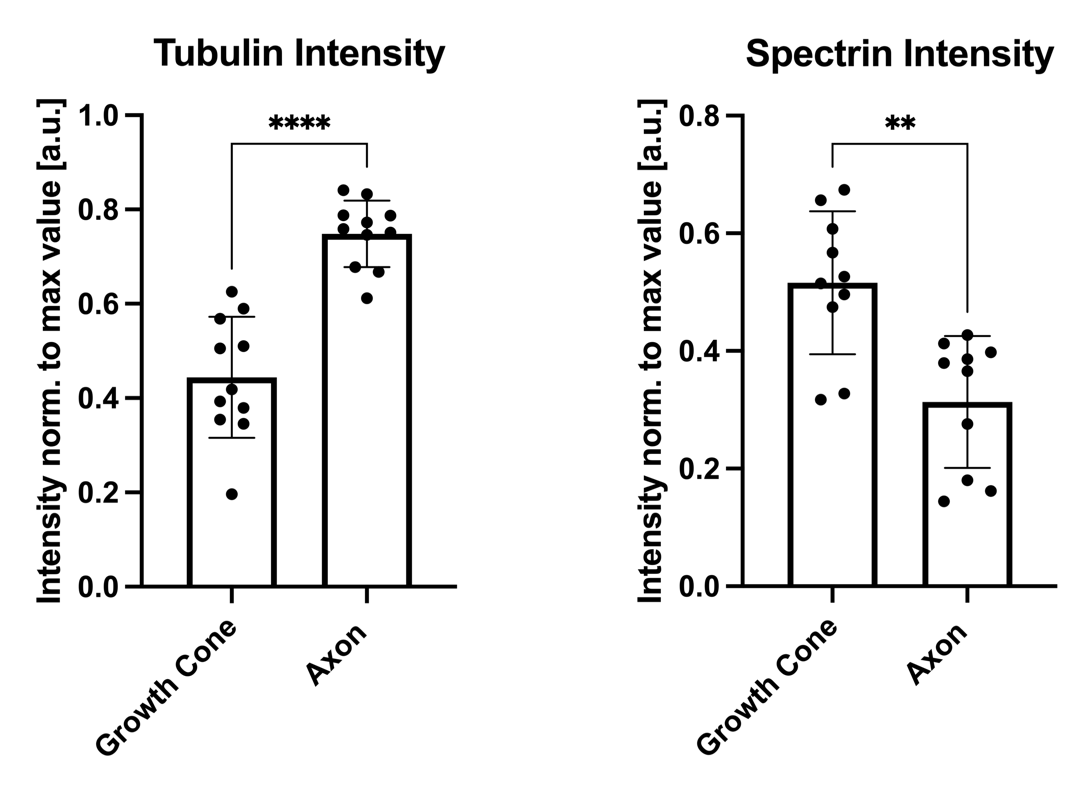


**Supplemental Figure 1. Distribution of βII-spectrin and βIII-tubulin along the axon and the growth cone in non-axotomized rat cortical neurons.** Quantification of 50 µm long line intensity scans along the axon, starting from the growth cone tip. Due to the mean growth cone length, the first 7 µm of the line intensities represent the GCs, whereas the other 7 – 50 µm represent the axons. βII-spectrin-fluorescence intensity peaks at the growth cone, whereas the βIII-tubulin signal is lower compared to the axon. Data is represented as mean ± SD. At least 4 biological replicates were analyzed, 11 axons were analyzed in total


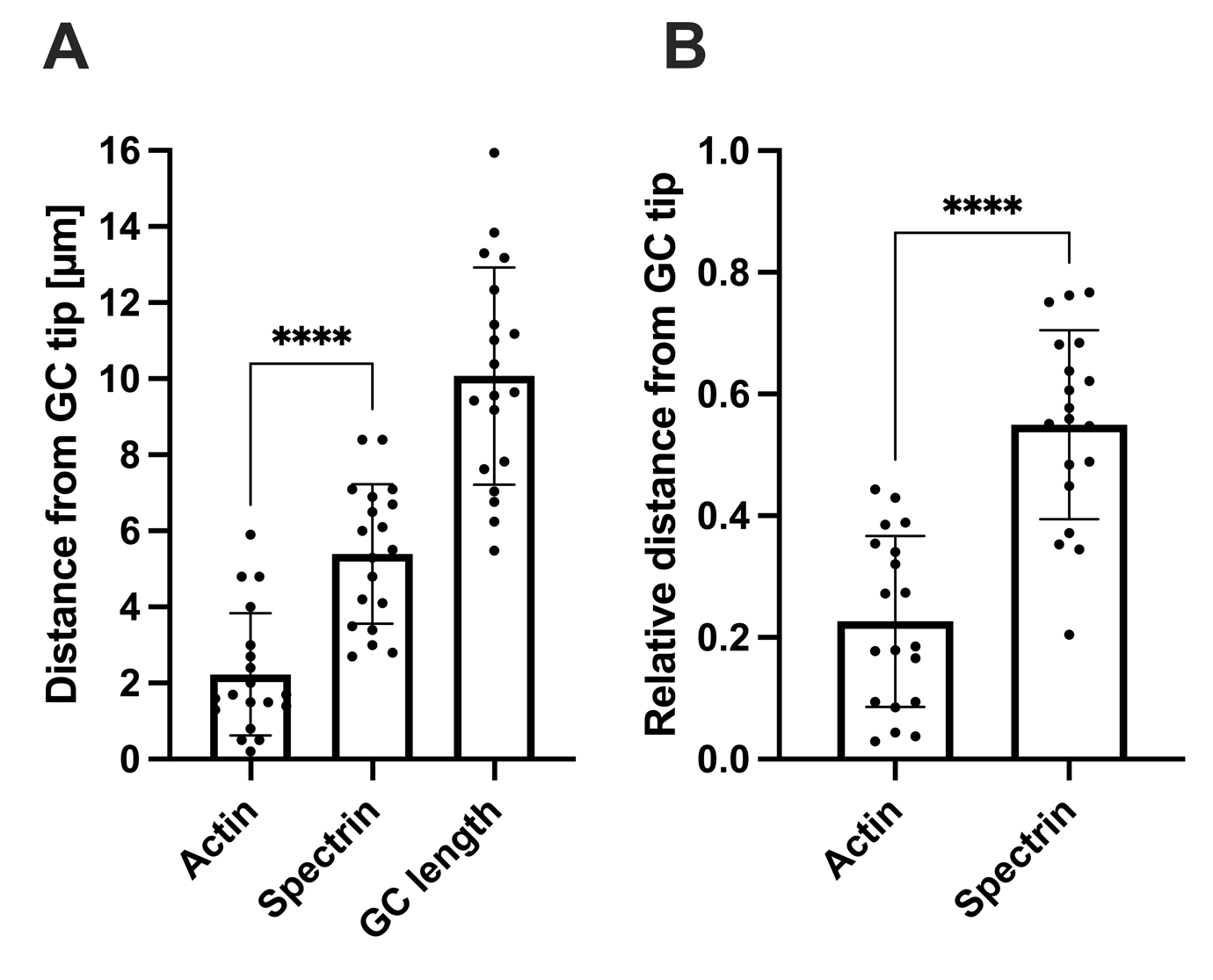


**Supplemental Figure 2. Localization of actin and spectrin fluorescence intensity peaks along the GC in non-axotomized rat cortical neurons.** Quantification of line intensity scans for spectrin and actin along the GC, starting from the growth cone tip. Intensity values were averaged for every 200 nm and the distance from the GC tip of the respective maximum intensity peak for spectrin and actin was noted. In **A**, the measured GC lengths are also displayed. **B** shows the distance of respective spectrin and actin intensity peaks relative to the individual GC length. The actin signal peaks closer to the GC tip compared to the spectrin signal. At least 2 biological replicates were analyzed, 19 GC were analyzed in total.


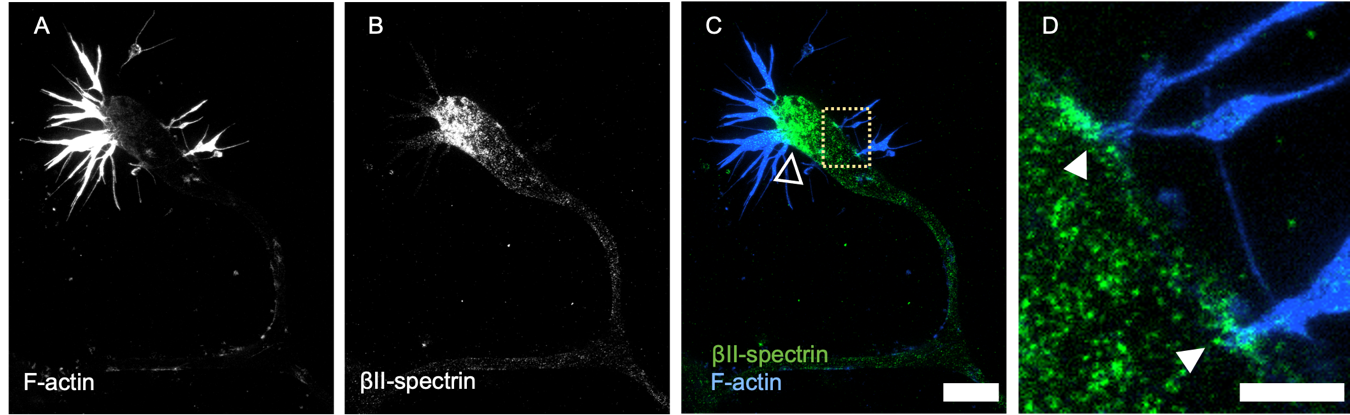


**Supplemental Figure 3. Exemplary STED image of a growth cone stained for F-actin and spectrin.** Spectrin is enriched in the growth cone, in particular close to the base of filopodia (open arrowhead). **D** shows an enlarged version of **C**, closed arrowheads point to enriched spectrin at the base of filopodia. This proximity was seen in some axons. Scale bar 4 µm in **A-C** and 1 µm in **D**.


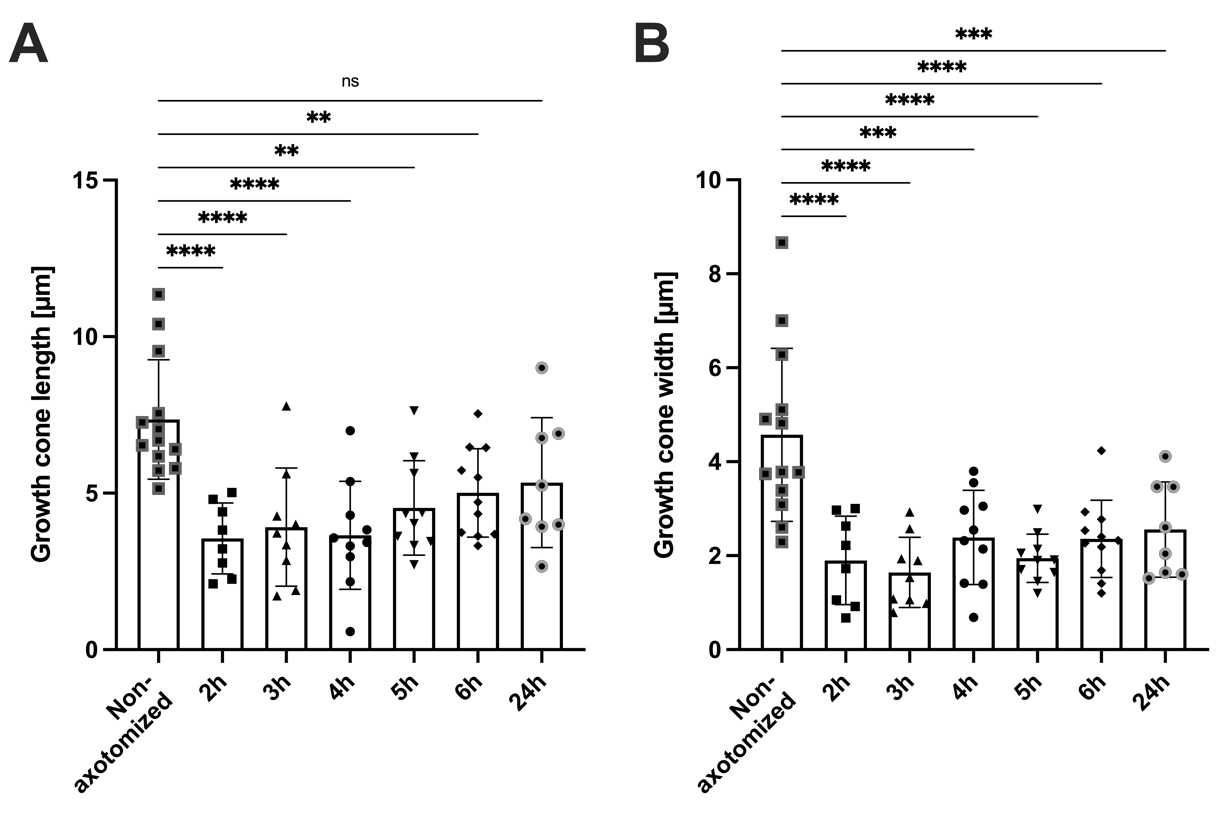


**Supplemental Figure 4. Difference of GC length and width of non-axotomized and regenerating axons.** Comparison of growth cone length **(A)**, and growth cone width **(B)** of different time points after axotomy and non-axotomized axons, quantified in spectrin-stained axons. The growth cone length and width of regenerating axons are significantly reduced compared to non-axotomized axons, except for the growth cone width of the 24-hour time point. Bars represent mean ± SD; one-way ANOVA, followed by Dunnett’s multiple comparisons test, was performed. At least 8 axons were analyzed per condition, n=3, 67 axons were analyzed in total. **(A)** The length of regenerating GCs at 2 hours after axotomy was decreased two-fold compared to non-axotomized axons. GC length was still significantly shorter up to 6 hours, but not 24 hours after axotomy (2 hours: 3.6 µm ± 0.4 µm, 6 hours: 5.0 µm ± 0.4 µm, 24 hours: 5.3 µm ± 0.7 µm, and non-axotomized axons: 7.4 µm ± 0.5 µm). **(B)** The width of regenerating GCs was reduced by almost 2.5-times at 2 hours after axotomy, compared to non-axotomized axons. This trend did not normalize over 24 hours, where GC width was still decreased by 1.8-times compared to the non-axotomized group (2 hours: 1.9 µm ± 0.3 µm, 24 hours: 2.6 µm ± 0.4 µm, and non-axotomized axons: 4.6 µm ± 0.5 µm).


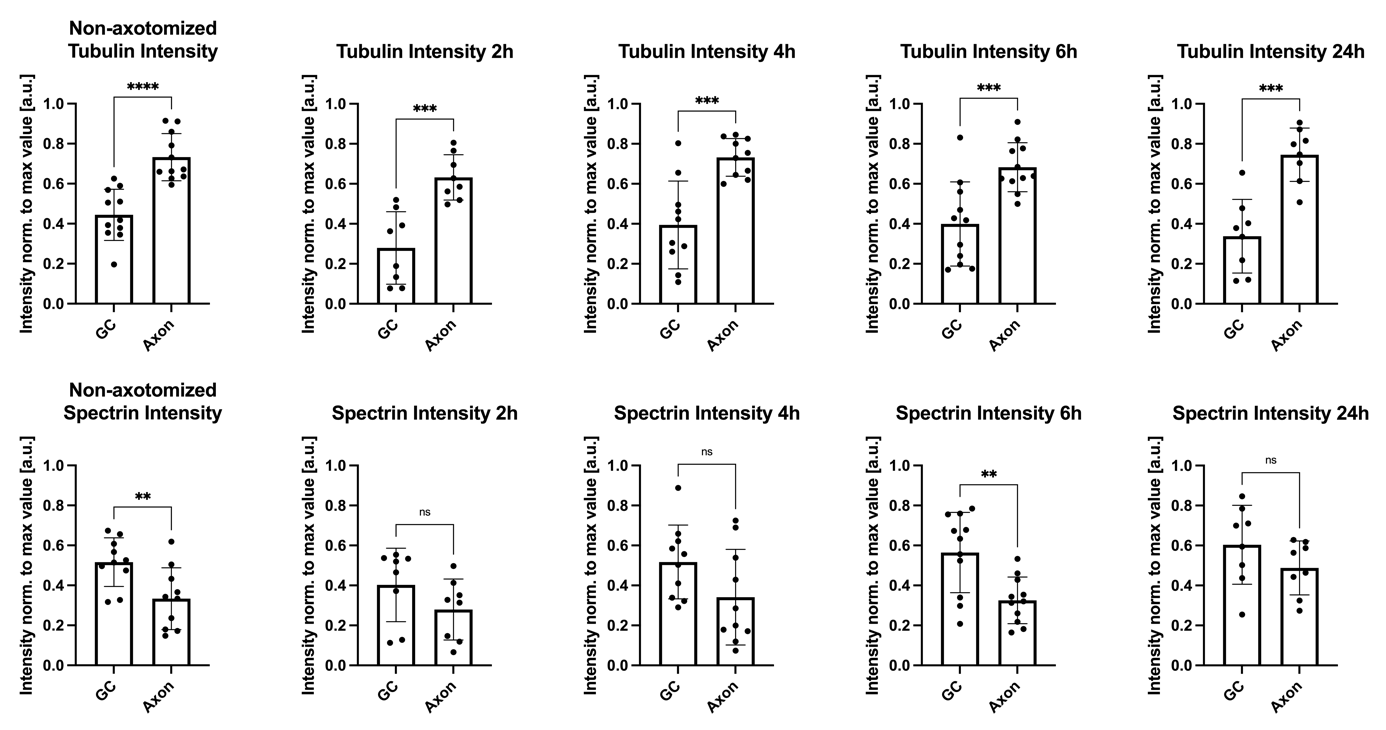


**Supplemental Figure 5.** **Distribution of βII-spectrin and βIII-tubulin along the axon and the growth cone in non-axotomized rat cortical neurons**. Quantification of line intensity scans along the axon of sampled groups at different time points after axotomy and before axotomy, starting from the growth cone tip and the first 10 µm of the axon. Intensity values were averaged for every 1 µm and normalized to the maximum intensity. βII-Spectrin-fluorescence intensity peaks at the growth cone of non-axotomized and 6-hour time point, whereas the βIII-tubulin signal is lower in the GC in all groups, compared to the axon. Data is represented as mean ± SD. At least 4 biological replicates were analyzed, 47 axons were analyzed in total.


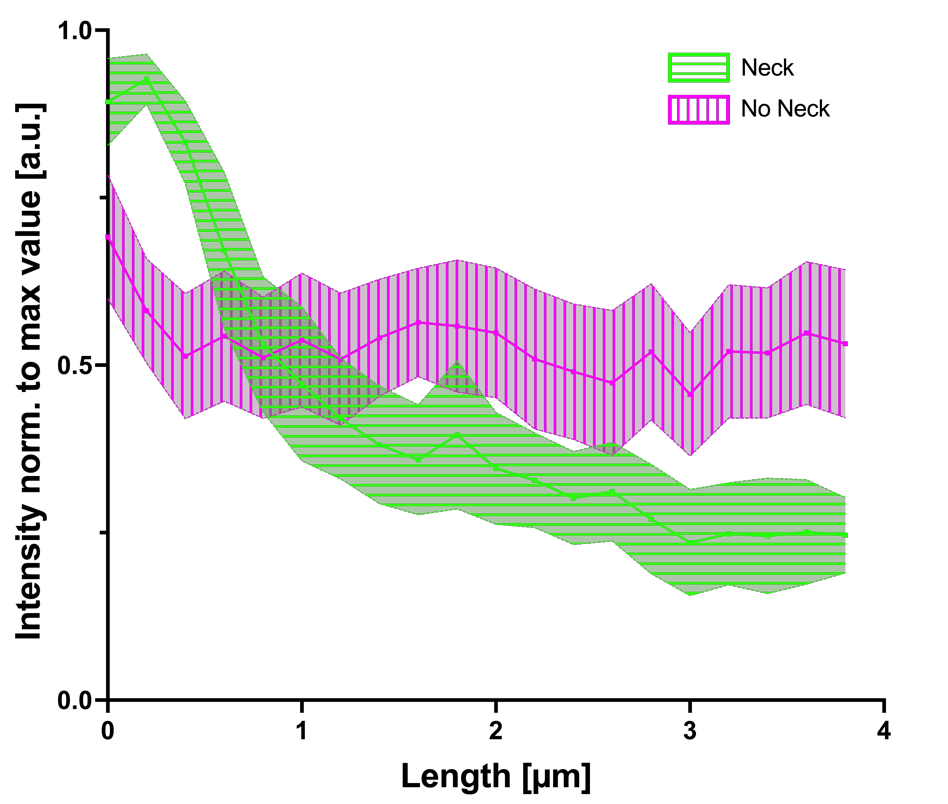


**Supplemental Figure 6. Comparison of spectrin intensity distribution in regenerating axons with or without spectrin neck.** Line intensity scans were performed along the first 4 µm of axon adjacent to the GC. All time points after axotomy are combined in this graph. A spectrin neck was defined as an at least 600 nm long increase of spectrin signal, relative to the more proximal axon, at the axonal segment directly connected to the GC, not longer than 3 micrometers. The increase had to be higher than the 75^th^ percentile of the measured 4 µm, and the first micrometer had to have 1.5 times higher intensity values than the 2^nd^ – 4^th^ micrometer. Axons displaying spectrin necks showed higher values of spectrin intensity in the first micrometer, compared to axons without spectrin necks. At least 21 axons were analyzed per condition, n=3, 56 axons were analyzed in total.


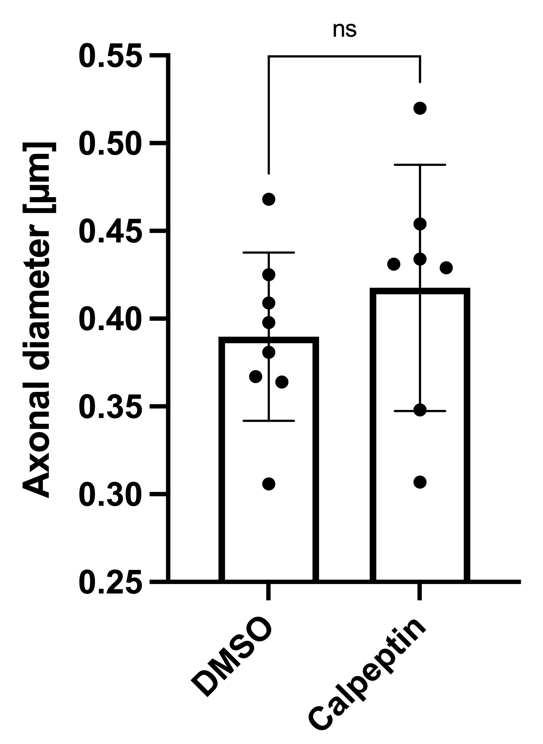


**Supplemental Figure 7. Diameter of the first 10 µm of axon adjacent to the growth cone in calpeptin or DMSO treated axons.** Cells were cultured for 11 days. On DIV 11, calpeptin or DMSO was administered into the axonal compartment for 1 hour and axotomized afterward. Regenerating axons were fixed 120 minutes later and stained for spectrin and tubulin. The axonal diameter was then measured at 10 µm from the GC. Data is given as mean ± SD. At least 7 Axons were analyzed per condition, n=3, 15 axons were analyzed in total. No significant difference was detected according to unpaired t test.


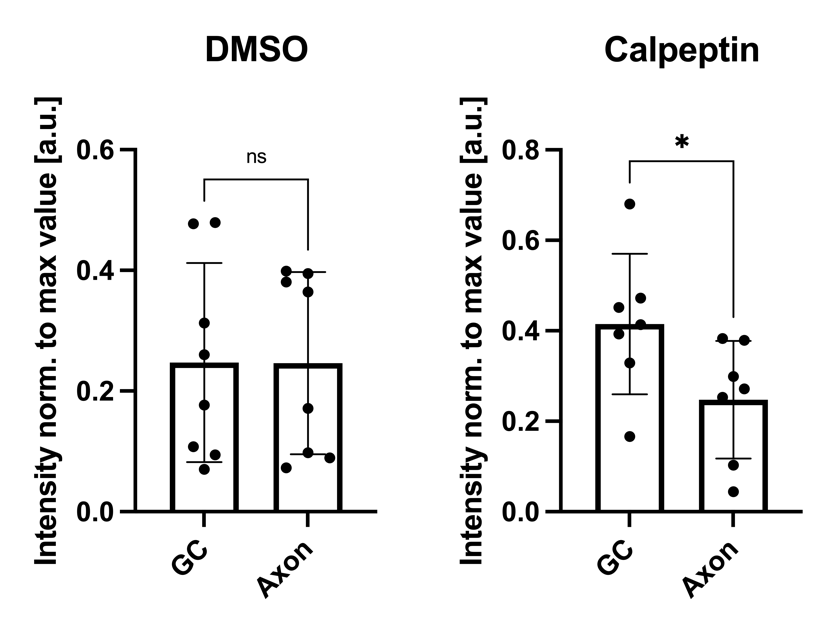


**Supplemental Figure 8. Distribution of βII-spectrin along the axon and the growth cone in rat cortical neurons treated with DMSO or calpeptin.** Quantification of line intensity scans along the axon and GC of sampled groups treated with DMSO or Calpeptin at 2 hours after axotomy, starting from the growth cone tip and the first 10 µm of the axon. βII-Spectrin-fluorescence intensity peaks at the growth cone of calpeptin treated neurons, compared to the axon. In DMSO treated neurons, no difference in spectrin intensity between GC and axon was detected. Data is represented as mean ± SD. At least 3 biological replicates were analyzed, 15 axons were analyzed in total.
